# Labeling galectin-3 for the assessment of myocardial infarction in rats

**DOI:** 10.1186/s13550-014-0075-x

**Published:** 2014-12-14

**Authors:** Teresa Arias, Artiom Petrov, Jiqiu Chen, Hans de Haas, Carlos Pérez-Medina, Gustav J Strijkers, Roger J Hajjar, Zahi A Fayad, Valentín Fuster, Jagat Narula

**Affiliations:** Zena and Michael A. Wiener Cardiovascular Institute, Icahn School of Medicine at Mount Sinai, One Gustave L. Levy Place, Box 1030, New York, NY 10029 USA; Centro Nacional de Investigaciones Cardiovasculares (CNIC), Melchor Fernández Almagro, 3, Madrid, 28029 Spain; Department of Biomedical Engineering and Physics, Academic Medical Center (AMC), PO Box 22700, 1100 DE Amsterdam, The Netherlands

**Keywords:** Galectin-3, Myocardial infarction, Myocardial remodeling, Molecular imaging, SPECT

## Abstract

**Background:**

Galectin-3 is a ß-galactoside-binding lectin expressed in most of tissues in normal conditions and overexpressed in myocardium from early stages of heart failure (HF). It is an established biomarker associated with extracellular matrix (ECM) turnover during myocardial remodeling. The aim of this study is to test the ability of ^123^I-galectin-3 (IG3) to assess cardiac remodeling in a model of myocardial infarction (MI) using imaging techniques.

**Methods:**

Recombinant galectin-3 was labeled with iodine-123 and *in vitro* binding assays were conducted to test ^123^I-galectin-3 ability to bind to ECM targets. For *in vivo* studies, a rat model of induced-MI was used. Animals were subjected to magnetic resonance and micro-SPETC/micro-CT imaging two (2 W-MI) or four (4 W-MI) weeks after MI. Sham rats were used as controls. Pharmacokinetic, biodistribution, and histological studies were also performed after intravenous administration of IG3.

**Results:**

*In vitro* studies revealed that IG3 shows higher binding affinity (measured as counts per minute, cpm) (*p* < 0.05) to laminin (2.45 ± 1.67 cpm), fibronectin (4.72 ± 1.95 cpm), and collagen type I (1.88 ± 0.53 cpm) compared to bovine serum albumin (BSA) (0.88 ± 0.31 cpm). Myocardial quantitative IG3 uptake (%ID/g) was higher (*p* < 0.01) in the infarct of 2 W-MI rats (0.15 ± 0.04%) compared to control (0.05 ± 0.03%). IG3 infarct uptake correlates with the extent of scar (*r*_s_ = 1, *p* = 0.017). Total collagen deposition in the infarct (percentage area) was higher (*p* < 0.0001) at 2 W-MI (24.2 ± 5.1%) and 4 W-MI (30.4 ± 7.5%) compared to control (1.9 ± 1.1%). However, thick collagen content in the infarct (square micrometer stained) was higher at 4 W-MI (20.5 ± 11.2 μm^2^) compared to control (4.7 ± 2.0 μm^2^, *p* < 0.001) and 2 W-MI (10.6 ± 5.1 μm^2^, *p* < 0.05).

**Conclusions:**

This study shows, although preliminary, enough data to consider IG3 as a potential contrast agent for imaging of myocardial interstitial changes in rats after MI. Labeling strategies need to be sought to improve *in vivo* IG3 imaging, and if proven, galectin-3 might be used as an imaging tool for the assessment and treatment of MI patients.

## Background

Myocardial remodeling is one of the principal characteristics of heart failure (HF) [1,2], which remains a major cause of morbidity and mortality, both in the high and low income countries [3]. Myocardial remodeling is the result of alterations in the cellular and subcellular elements of cardiomyocyte and non-cardiomyocyte components of the myocardium and results primarily from the excessive accumulation of collagen in interstitial and perivascular regions [1]. Initially, the alterations associated with remodeling appear to compensate for the damage whereas its persistence over time causes progressive adverse remodeling and results in HF regardless of the etiology [4]. Given that the progressive myocardial fibrosis produces abnormalities that affect cardiac function and electrical activity of the heart and plays an important role in the development of HF, it is of great importance to develop novel circulating biomarkers and noninvasive imaging strategies that accurately define the evolution of myocardial fibrosis. In this context, galectin-3 has been proposed as a peripheral biomarker that has been related with inflammation and fibrosis underlying adverse cardiac remodeling [5-8]. Galectin-3 is a 29- to 35-KDa ß-galactoside-binding animal lectin [9] expressed by activated macrophages, eosinophils, neutrophils, and mast cells [10]. There are many ligands for galectin-3, some of them are extracellular matrix components like laminin or fibronectin [9-11]. Galectin-3 is present in most tissues in normal conditions and overexpressed in myocardium from the early stages of HF [12], especially post-myocardial infarction (MI) [13,14]. Galectin-3 induces myocardial fibroblast proliferation, collagen deposition, and ventricular dysfunction in rats [15]; galectin-3 binding sites are abundantly expressed on rat cardiac fibroblasts and within the extracellular matrix (ECM) [15]. Therefore, galectin-3 has been proposed as a biomarker associated with ECM turnover in the setting of HF [16,17]. The serum levels of galectin-3 in HF patients are significantly correlated with other established biomarkers of ECM turnover such as type III aminoterminal propeptide of procollagen (PIIINP), matrix metalloprotease-2 (MMP-2), and tissue inhibitor of metalloprotease-1 (TIMP-1), as also with the New York Heart Association (NYHA) functional class [18].

Because binding sites are excessively expressed in ECM and on proliferating myofibroblasts, we investigated the suitability of iodine-123-labeled galectin-3 (IG3) as a single-photon emission computed tomography (SPECT) probe for myocardial remodeling imaging in a rodent model of post-MI HF. Animal studies were conducted in rats 2 and 4 weeks after MI based on our previous studies with mice [19,20], which demonstrated a peak in fibrosis-tracer uptake at those time points. Prior to animal studies, chemical and binding properties of IG3 were tested *in vitro* using solid-phase assays.

## Methods

### Galectin-3 radiolabeling

Galectin-3 radiolabeling was performed using carrier-free Na^123^I (Nordion, Ottawa, ON, Canada) and iodotubes (Pierce, Rockford, IL, USA). Briefly, aliquots of 150 μg (2 mg/ml) of recombinant human galectin-3 (Peprotech, Rocky Hill, NJ, USA) were incubated with 1 mCi of Na^123^I for 15 min at room temperature (RT). Labeled protein was separated from free iodine species by gel filtration on Sephadex G-25 columns (GE Healthcare Bio-Sciences, Pittsburgh, PA, USA). Afterwards, activity was checked by gamma counting and protein concentration quantified by UV spectrophotometry. Radiochemical purity and stability of the tracer was determined by thin layer chromatography (TLC) on silica gel.

### Solid-phase radiolabeling binding assay

*In vitro* solid-phase radiolabeling binding assays were carried out to check whether labeling altered the galectin-3 structure and to confirm the ability of IG3 to bind to ECM targets. Briefly, U-bottom 96-well plates (Becton Dickinson Labware, Mississauga, ON, Canada), previously coated with bovine serum albumin (BSA) at 5%, were incubated (overnight at 4°C) with galectin-3 targets, namely fibronectin, laminin [9-11], and collagen type I [21], and with BSA as control (50 μg/ml in Phosphate Buffered Saline (PBS)). After incubation with IG3 for 5 h at RT, wells were washed and the bottoms cut off to measure bound radioactivity using a gamma counter (1480 Wizard 3, Perkinelmer, Waltham, MA, USA).

### Animal model of MI

All procedures followed the recommendations of the Guide for the Care and Use of Laboratory Animals (Department of Health and Human Services publication no. NIH 78–23, 1996) and approved by the Icahn School of Medicine at Mount Sinai Animal Care and Use Committee.

Briefly, male Sprague–Dawley rats (200 to 250 g) underwent permanent left anterior descending artery ligation (LAD) (*n* = 6) as previously described [2,22]. Sham animals underwent thoracotomy without permanent LAD (*n* = 6).

Animals were divided into two groups at 2 weeks (*n* = 3) and 4 weeks (*n* = 3) post-MI (2 W-MI and 4 W-MI, respectively), in order to evaluate changes in myocardial remodeling after MI using IG3 imaging.

### Pharmacokinetics and biodistribution studies

After intravenous administration of IG3, blood samples were collected at different time points (15 min, 30 min, 1 h, 2 h, 3 h, and 4 h). Duplicates of blood samples were analyzed by gamma counting for radioactivity and TLC for chemical stability.

We undertook biodistribution and pharmacokinetics studies for IG3 from John CM et al. [23] and decided to explant the organs 4 h after injection, washed in PBS, and collected to analyze radioactivity tissue distribution by gamma counting.

### Cardiac magnetic resonance

To assess ventricular function and myocardial infarct size, rats underwent cardiac magnetic resonance (CMR) 2 days before nuclear imaging.

Rats were sedated with 1.5% to 2% of isoflurane in oxygen (0.6 l/min). Respiratory rate was monitored using a magnetic resonance (MR)-compatible monitoring system. Gd-DTPA (0.6 mmol/Kg, Magnevist®) was injected via tail vein, as contrast agent, 20 min before full-volume left ventricle and infarct imaging.

A 7 T horizontal bore animal MR scanner (Bruker BioSpin GmbH, Ettlingen, Germany) was used with a retrospectively self-gated protocol (IntraGate) as previously described [2,22]. Briefly, full-volume heart imaging acquired 20 min after Gd-DTPA injection was used for left ventricle (LV) function and infarct volume measures. Sequence parameters were as follows: radiofrequency pulse (RF): 1 ms sinc pulse (10 lobes, bandwidth: 20 kHz); slab thickness: 10 mm, 10 mm; FOV: 50 × 50 × (18 to 14) mm^3^; Nx × Ny × Nz: 128 × 128 × (12 to 9); spatial resolution: 0.312 × 0.312 × 1.5 mm/pixel; TE: 2 ms; TR: 8 ms; number of repetitions (NR): 50 and flip angle α: 30°; On navigator slice: RF pulse: 1 ms Gauss pulse (bandwidth: 2.740 kHz); slice thickness: 5 mm and flip angle α^nav^: 8°. No more than 70% of all k-lines were used for the reconstruction of 16 cardiac time frames to avoid artifacts from respiratory movement. Total scan time was approximately 8 to 10 min (Ny × Nz × TR/NR). The effective number of averages was approximately 2.5 (70% × NR/6).

Analysis of global LV function and infarct volume was calculated using Segment software (Segment v1.9, Medviso, Sweden). Myocardium segmentation was performed among at least seven LV short-axis slices outlining both endocardial and epicardial borders in all the cardiac frames. Infarct area was defined as the bright area on short-axis images with the largest area of hyperenhancement in both end-systole and end-diastole frames.

### Micro-SPECT/micro-CT imaging with IG3

Radionuclide imaging was performed using a dual-head micro-single-photon emission computed tomography (μSPECT) gamma camera with micro-computed tomography (μCT) (X-SPECT, Gamma Medica Inc-Siemens, Northridge, CA, USA). Each animal was injected with approximately 1 mCi of IG3 (150 μg of protein) via tail vein.

Based on our previous experience and others’ reports [19,20,24], *in vivo* heart images were acquired 4 h after injection under isoflurane anesthesia as described previously [25]. Subsequently, animals were euthanized by carbon dioxide asphyxiation and animal myocardium was explanted for static *ex vivo* images acquired for 15 min using a low-energy, high-resolution, parallel-hole collimator. Then, organ samples were weighed and the percentage of injected dose per gram (%ID/g) of tissue determined by gamma counting.

### Histopathological characterization of myocardial specimens

Following imaging studies, cardiac specimens where divided into three parts (apex/infarct, MI border and MI remote tissue), fixed, and embedded in OCT for histopathological studies.

Heart cryosections were used to study myocardial fibrosis content using Picrosirius Red Staining (Spectrum Chemical, New Brunswick, NJ, USA) as previously described [26]. Three sections of each sample were stained and digitally recorded by Zeiss Axioplan2 microscope and Zeiss AxioVision software (Micro-optik). Total collagen content was recorded using a bright field light whereas quality of collagen fibers was further studied using polarized light which allows the qualitative quantification of the mature, thick fibers as orange and red fibers, and the newly formed, thin as green and yellow [27]. Collagen deposition was analyzed by Image J software and presented as percent of area stained (mean ± SD) for total collagen content or square micrometer stained (mean ± SD) for the different types of collagen fibers.

### Statistical analysis

Quantitative radiotracer uptake *in vitro* was calculated as counts per minute (cpm) bound to immobilized proteins and as percent total injected dose per gram (%ID/g) for the tissue. To determine statistical significance of differences in quantitative *in vitro* data, unpaired *t*-test analysis was performed. To test significant difference among animal groups, one-way analysis of variance followed by *post hoc* Bonferroni test for multiple comparisons was used. The relation between heart uptake and infarct size was tested calculating Spearman correlation coefficient. *p* values < 0.05 were considered statistically significant.

## Results

### Radiolabeling of galectin-3

**Figure 1:**
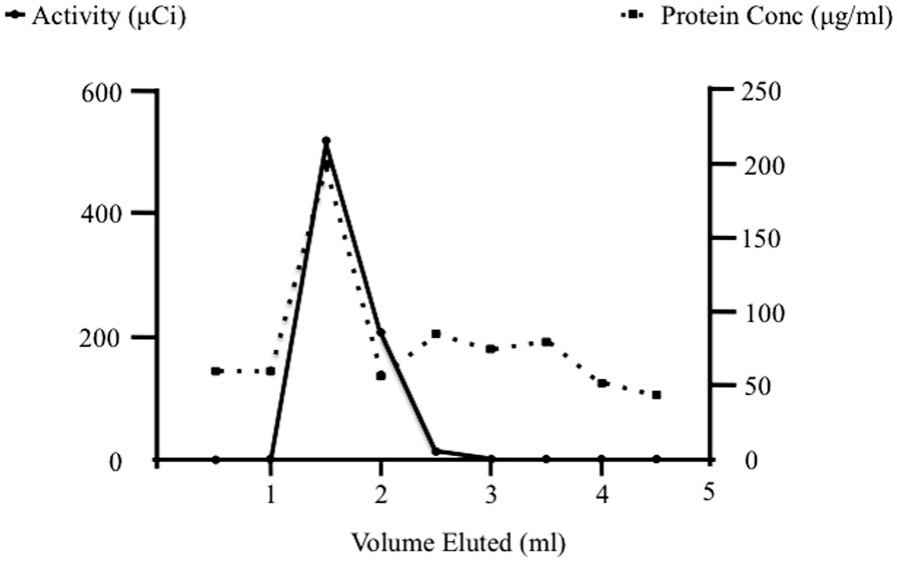
**IG3 purification on Sephadex G-25 after labeling.** IG3 specific activity (μCi) checked by gamma counting and protein concentration (μg/ml) quantified by UV spectrophotometry.

Recombinant human galectin-3 was successfully radiolabeled with Na^123^I (Figure 1). The total IG3 eluted between 1 and 2 ml and the radiochemical yield was > 90%. The radiochemical purity of IG3 was > 93% as assessed by radio-TLC. Chemical stability of the tracer in PBS was analyzed by radio-TLC at 4 h and 24 h after labeling. No significant change was observed at these two time points (90.4% and 90.1% of the main fraction remained intact 4 h and 24 h after labeling, respectively), suggesting a good chemical stability.

### IG3 *in vitro* binding assays

**Figure 2:**
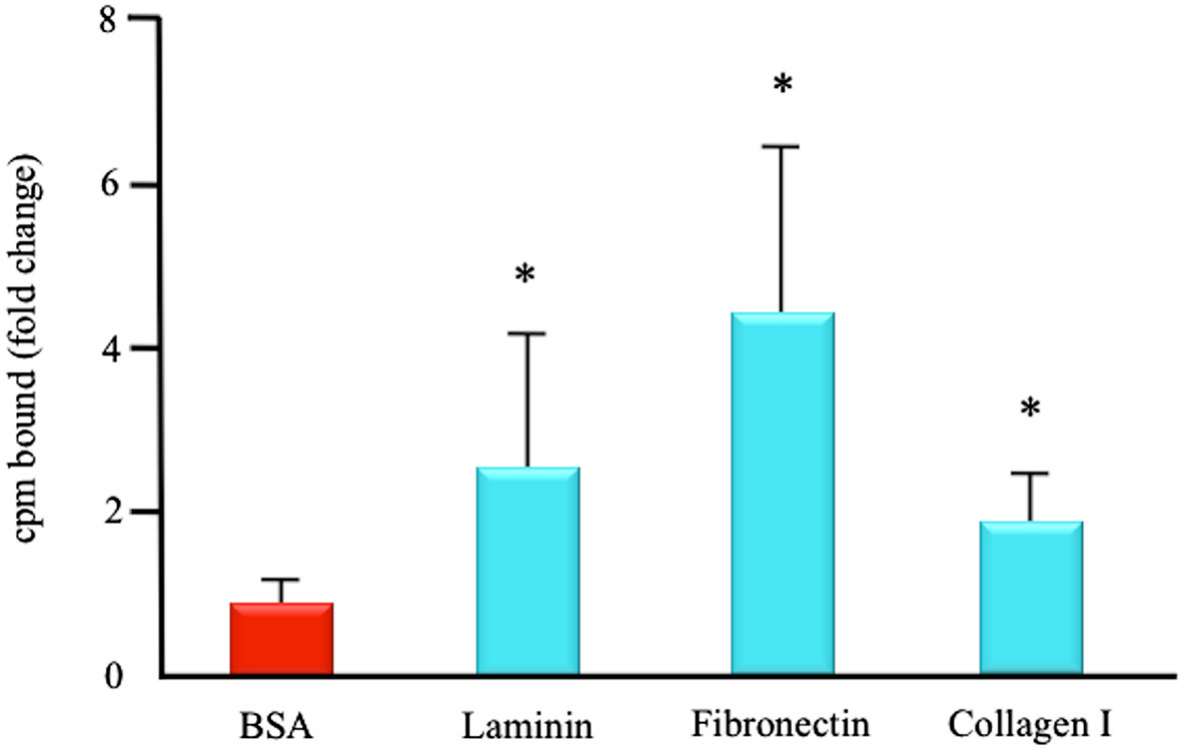
**IG3** ***in vitro*** **binding experiments.** Solid-phase radiolabeling binding assays to test IG3 ability to bind to its targets. Values are given as mean ± SD of duplicates measurements from seven independent experiments. *p < 0.05 vs. BSA. cmp, counts per minute.

Five hours after incubation with IG3 at RT, the bound radioactivity (cpm bound, mean ± SD) was significantly higher (*p* < 0.05) in those wells pre-coated with laminin (2.45 ± 1.67 cpm), fibronectin (4.72 ± 1.95 cpm), and collagen type I (1.88 ± 0.53 cpm) compared to control, BSA (0.88 ± 0.31 cpm) as shown in Figure 2. IG3 binding to ECM components was also observed pre-coating the wells at lower concentrations (10 μg/ml), showing a significant concentration-dependent binding of IG3 to laminin, fibronectin, and collagen type I (data not shown).

### IG3 *in vivo* stability

*In vivo* metabolic stability of IG3 was assessed using blood and heart samples 4 h after IG3 injection using TLC. Unlike the results observed in PBS, 28% and 40% of the main IG3 fraction remained intact in blood and homogenized heart extracts, respectively, pointing to *in vivo* degradation of the compound.

### IG3 biodistribution and organ uptake

**Figure 3:**
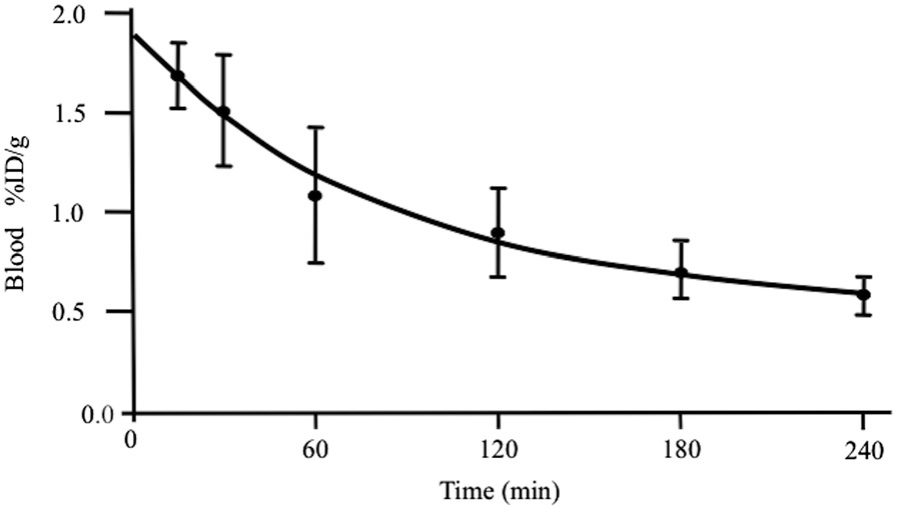
**IG3 blood uptake at different times after i.v. injection.** After IG3 injection, specific activity of blood samples was gamma counted. Values are given as %ID/g (mean ± SD) of duplicates measurements from twelve independent experiments. IG3 uptake decreased in a time-dependent manner (*p* for trend < 0.0001). % ID/g, percent injected dose per gram.

Blood pharmacokinetics of IG3 (Figure 3) showed a clearance in a time-dependent manner (*p* for trend < 0.0001); IG3 in blood decreased by 50% 2 h after injection and by 75% at 4 h.

IG3 biodistribution was examined in all 12 rats. Organ uptake and heart distribution of IG3 4 h after i.v. administration are shown in Table 1. Thyroids showed the highest uptake in the three groups, followed by the kidney, spleen, liver, and lung. However, no significant differences were observed in IG3 biodistribution between groups in the remaining vital organs, muscle, or bone.

**Table 1:** **Organ biodistribution of IG3**

	**Control**	**2 W-MI**	**4 W-MI**
MI infarct (apex)	0.058 ± 0.039	0.151 ± 0.047^†^	0.071 ± 0.059
MI border	0.047 ± 0.028	0.110 ± 0.021^*^	0.056 ± 0.044
MI remote	0.045 ± 0.033	0.108 ± 0.026^*^	0.055 ± 0.044
Lung	0.094 ± 0.034	0.194 ± 0.055	0.093 ± 0.073
Liver	0.236 ± 0.249	0.519 ± 0.095	0.192 ± 0.198
Spleen	0.079 ± 0.035	0.356 ± 0.041^Φ^	0.134 ± 0.118
Kidney	0.502 ± 0.101	0.784 ± 0.149	0.693 ± 0.367
Bone	0.057 ± 0.025	0.070 ± 0.013	0.053 ± 0.034
Thyroid	1.126 ± 1.409	1.326 ± 0.298	1.342 ± 0.223
Muscle	0.015 ± 0.004	0.021 ± 0.001	0.022 ± 0.013

Data are presented as %ID/g (mean ± SD) 4 h after injection.

%ID/g, percent injected dose per gram; MI, myocardial infarction; 2 W-MI, myocardial infarction 2 weeks old; 4 W-MI, myocardial infarction 4 weeks old.

*p < 0.05 control vs. 2 W-MI rats.

^†^ *p* < 0.01 control vs. 2 W-MI rats.

^Φ^ *p* = 0.0571 2 W-MI vs. control and 2 W-MI vs. 4 W-MI rats.

### IG3 myocardial uptake and imaging

LV ejection fraction (61.3 ± 7.4% vs. 54.0 ± 5.5%) and infarct size (16.5 ± 6.4% vs. 12.3 ± 2.2%) measured by CMR was similar in 2 W-MI and 4 W-MI animals, respectively.

**Figure 4:**
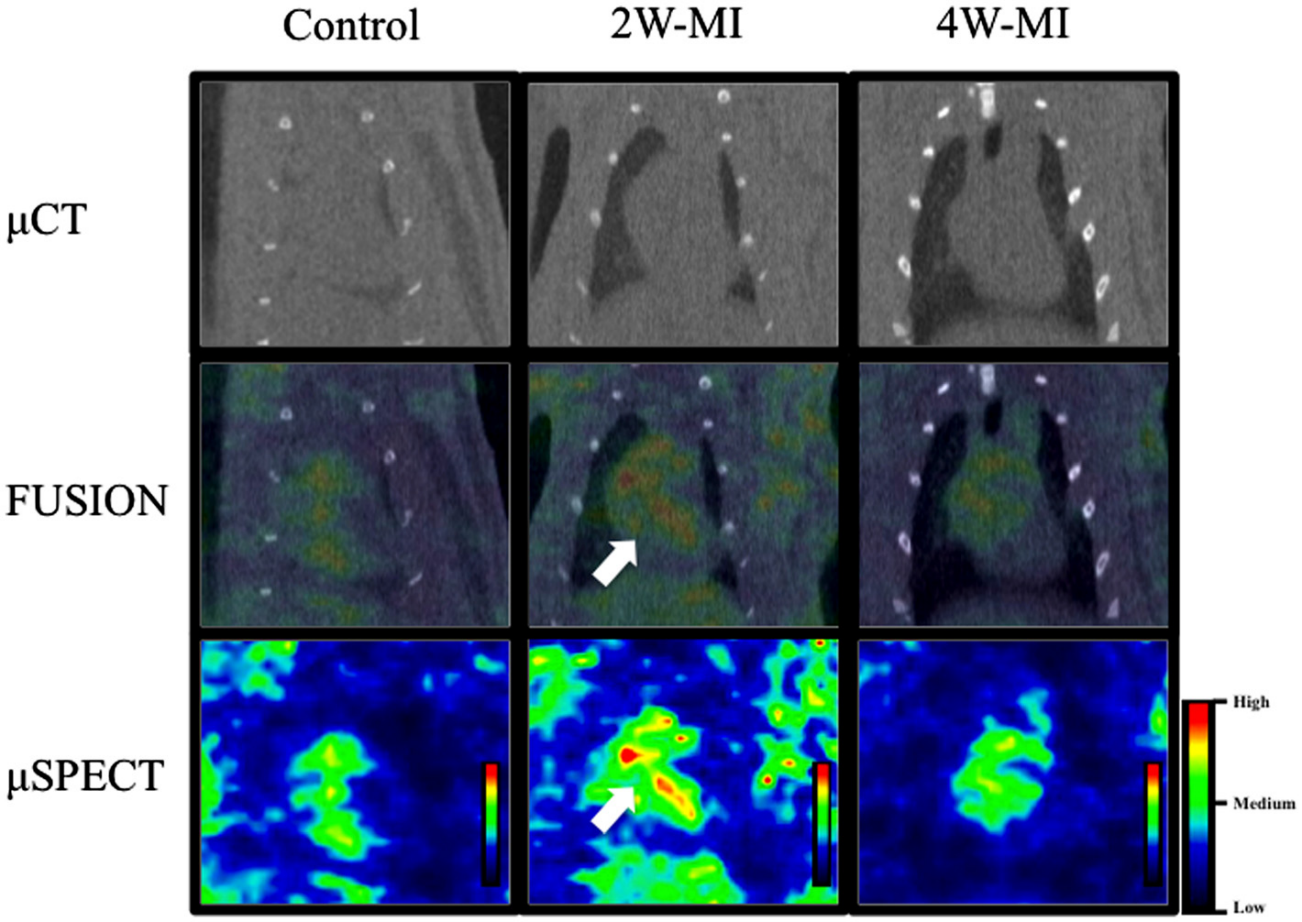
***In vivo*** **μCT, μSPECT, and fusion images in frontal projection.** Moderate IG3 uptake (white arrows) observed in the heart of 2 W-MI compared to control and 4 W-MI rats by μSPECT. Cardiac localization is confirmed in the fusion images. 2 W-MI, myocardial infarction 2 weeks old; 4 W-MI, myocardial infarction 4 weeks old; μCT, micro-computed tomography; μSPECT, micro-single-photon emission computed tomography.

**Figure 5:**
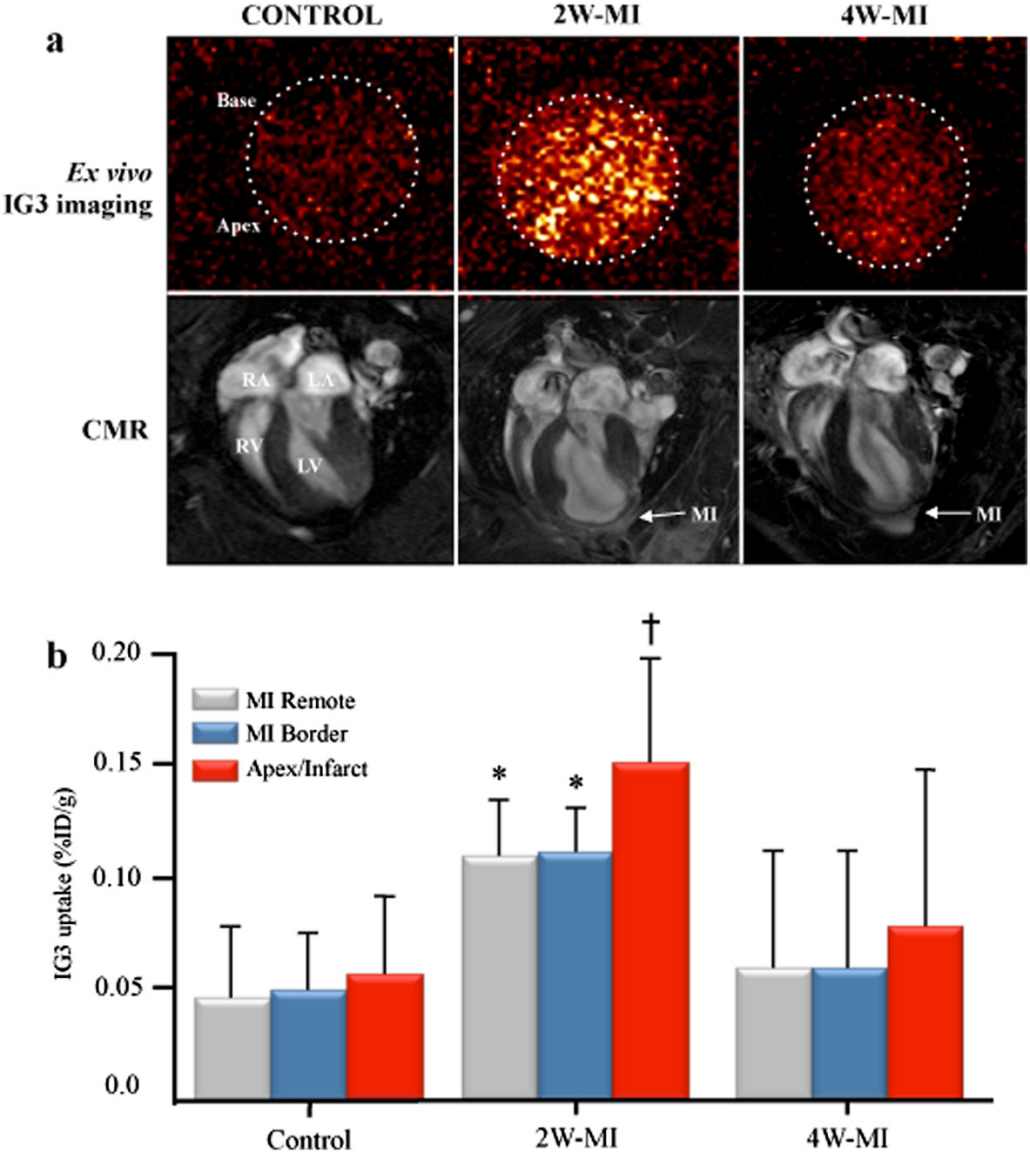
**IG3** ***ex vivo*** **heart imaging and uptake. (a)** Upper panel: *Ex vivo* heart imaging 4 h after IG3 administration; Lower panel: *In vivo* CMR: four chamber view after Gd-DTPA administration. Scar extension by LGE correlates with IG3 uptake (*r*_s_ = 1, *p* = 0.017). CMR, cardiac magnetic resonance; LA, left atrium; LGE, late gadolinium enhancement; LV, left ventricle; MI, myocardial infarction; μSPECT, micro-single-photon emission computed tomography; RA, right atrium; RV, right ventricle. **(b)** Explanted hearts were divided into three parts (apex/infarct, MI border, and MI remote) for gamma scintillation counting. Values are given as %ID/g (mean ± SD). *p < 0.05 2 W-MI vs. control. ^†^ *p* < 0.01 2 W-MI vs. control. %ID/g,% injected dose per gram; 2 W-MI, myocardial infarction 2 weeks old; 4 W-MI, myocardial infarction 4 weeks old.

*In vivo* radionuclide studies using μSPECT showed a moderate uptake of IG3 in the heart of 2 W-MI rats compared to control and 4 W-MI (Figure 4). Of interest, *ex vivo* planar images of the explanted heart confirmed increased uptake of IG3 in MI rats, especially in 2 W-MI rats compared to control and 4 W-MI rats (Figure 5).

IG3 uptake (%ID/g) in the heart was significantly (*p* < 0.01) higher in the apex/infarct of 2 W-MI rats (0.15 ± 0.05%) compared to the control animals (0.05 ± 0.04%). Heart uptake in MI remote (0.10 ± 0.03%) and MI border (0.11 ± 0.02%) areas of 2 W-MI rats was also significantly higher than control (0.04 ± 0.03% MI remote and 0.05 ± 0.03% MI border). There was no significant difference IG3 uptake in 4 W-MI rats compared to control and 2 W-MI rats (Figure 5).

In addition, quantitative apical uptake of IG3 (%ID/g) positively correlated with the CMR-verified extent of scar (%) defined by LGE (*r*_s_ = 1, *p* = 0.017).

### Collagen deposition in the myocardium

**Figure 6:**
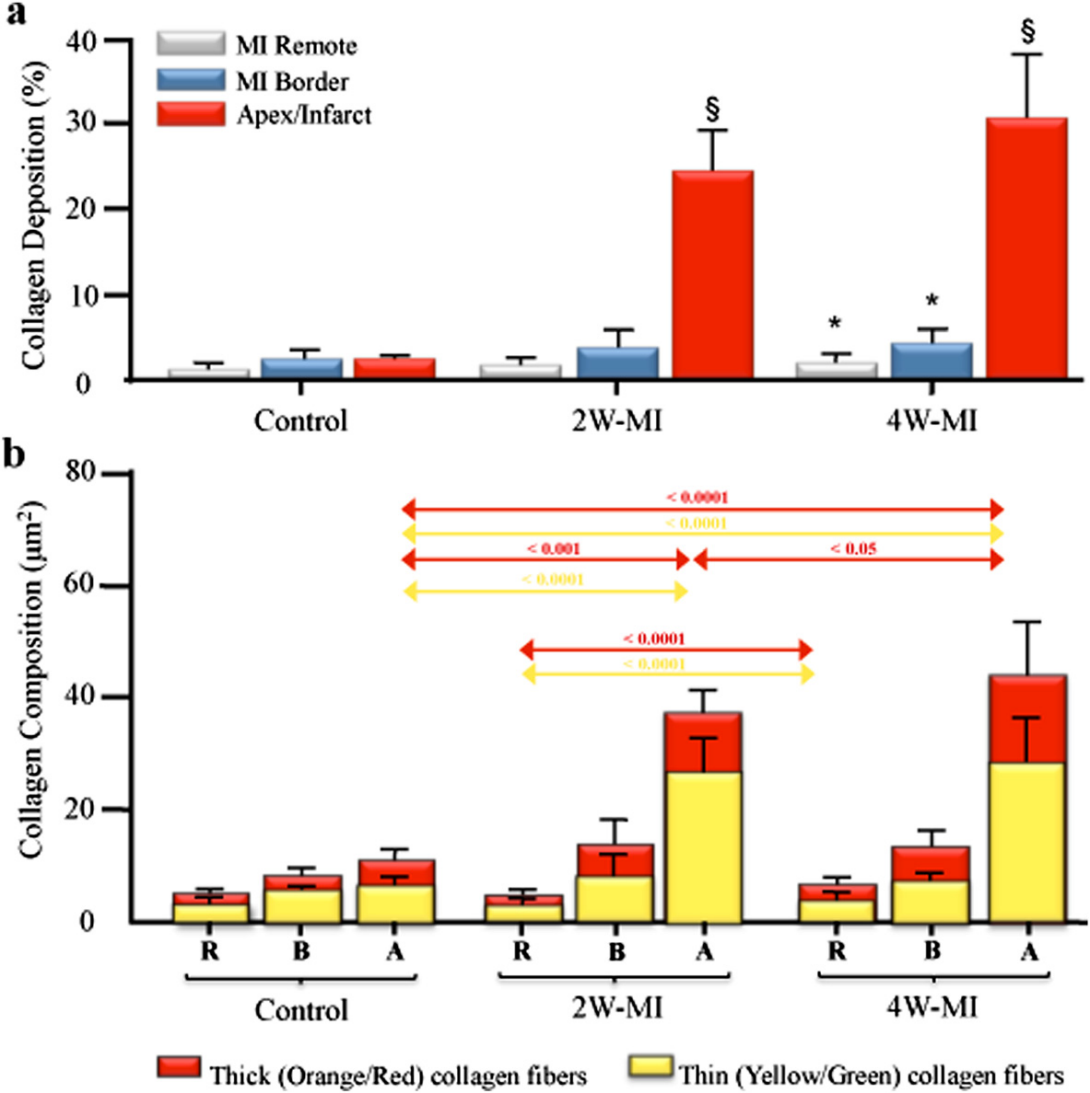
**Total collagen deposition and collagen composition in the myocardium. (a)** Total collagen deposition in the myocardium. Values are given as percent of area stained (mean ± SD). *p < 0.05 vs. control, ^§^ *p* < 0.0001 vs. control. **(b)** Collagen composition in the three heart regions. MI remote (R), MI border (B), and apical/infarct (A). Values are given as square micrometer stained (mean ± SD). 2 W-MI, myocardial infarction 2 weeks old; 4 W-MI, myocardial infarction 4 weeks old.

The analysis of collagen deposition by Picrosirius Red staining showed that the total amount of collagen at 2 and 4 weeks post-MI in the three different areas of the myocardium was similar (Figure 6a). However, the total amount of collagen in the infarcted (apical) region was significantly higher at 2 weeks (24.2 ± 5.1%) and 4 weeks (30.4 ± 7.5%) post-MI compared to control (1.9 ± 1.1%). No differences of total amount of collagen were observed in the border and remote areas of MI between control and 2 W-MI animals. Of interest, the total amount of collagen in those regions was significantly higher in 4 W-MI animals (MI border: 3.8 ± 2.0% and MI remote: 1.9 ± 1.2%) compared to control (MI border: 1.9 ± 1.5% and MI remote: 1.1 ± 0.7%).

When we analyzed by Picrosirius polarized light the quality of collagen fibers in the apex/infarct (Figure 6b), we found that the amount of mature thick fibers (orange and red) at 2 and 4 weeks post-MI was significantly higher than control animals as well as the amount of newly formed thin fibers (yellow and green). Interestingly, between 2 and 4 weeks post-MI, the only significant difference found was that the amount of mature collagen fibers at 4 weeks post-MI (20.5 ± 11.2 μm^2^) was higher than at 2 weeks (10.6 ± 5.1 μm^2^). In the MI border zone, the quality of collagen fibers was similar in all the groups (Figure 6b) and in the MI remote area (Figure 6b), the deposition of thick and thin collagen fibers in the 4 W-MI rats (2.4 ± 1.9 μm^2^ thick and 3.6 ± 1.6 μm^2^ thin) was higher compared to 2 W-MI rats (1.1 ± 0.7 μm^2^ thick and 2.4 ± 0.9 μm^2^ thin).

## Discussion

The current study presents a proof of principle that the radiolabeled IG3 could be employed to target the interstitial alterations that take place in the myocardium after an ischemic injury. These preliminary results demonstrate a significant increase of IG3 uptake in the myocardium of 2 W-MI rats compared to control rats.

### IG3 biodistribution

After successful labeling and confirming the chemical stability of the tracer at least for 24 h in PBS by TLC, we performed solid-phase *in vitro* binding assays (Figure 2) that demonstrated that IG3 retained the ability to bind to certain ECM components [10]. These results suggested that IG3 should be able to successfully bind to these ECM targets *in vivo*.

Interestingly, we observed in the animal studies that only 28% of the IG3 fraction in blood remained intact 4 h after injection suggesting degradation of the compound *in vivo*. The radioiodination of proteins using solid-phase oxidative approaches such as the Iodogen method offers a convenient route to radiolabel probes that usually affords iodinated histidine and tyrosine residues. These residues, however, are prone to deiodination after *in vivo* administration [28] and generally result in low label stability and loss of free iodide. Thus, the high thyroid uptake described points to deiodination of the tracer as the main metabolic transformation. In addition to the thyroid, the lungs, liver, and spleen showed high IG3 uptake suggesting, as described previously [23], that some organs may accumulate galectin-3 and serve as reservoirs for eventual systemic release or may participate in IG3 circulation removal. Interestingly, 4 h after injection, the intact IG3 fraction in the myocardium was 40%, significantly higher than that observed in the blood. This finding suggests that despite the substantial IG3 blood clearance and *in vivo* degradation, there seems to be a slight IG3 uptake in cardiac tissue that may be evidence of specific binding and sufficient for *ex vivo* planar imaging. In addition, IG3 heart uptake was significantly higher at 2 weeks post-MI compared to the control rats, whereas 4 weeks post-MI the IG3 uptake was completely resolved. These results agree with the pattern of thin collagen fiber production as previously described [19]; the decrease over time of fibrosis-tracer uptake in the infarct region correlated with the reduction of newly formed thin collagen fibers (yellow and green) [27]. Interestingly, this study also described that even though the total amount of collagen remained similar over time the thick collagen fibers (orange and red) increased over time, in agreement with our histological results (Figure 6b). Yet another study [29], described three overlapping phases of myocardial remodeling after injury in rodents: 1) Inflammatory (1 h to 8 h), Proliferative (48 h to 5 days), and 3) Maturation phase (5 days to 28 days). The peak expression of ECM components in the scar is during the proliferative phase whereas during maturation phase most of myofibroblast and endothelial cells resolve and the formation of a mature scar (comprised of dense cross-linked collagen fibers) enhances myocardial stiffness and thus eventually prompts to diastolic dysfunction and may lead to infarct expansion and systolic dysfunction.

Given that EMC components’ peak expression is within the first week after MI and decreases from then on, we hypothesize that the decrease of IG3 uptake observed 4 weeks after MI is related to a decrease in the amount of thin collagen fibers, myofibroblast, and the rest of ECM components. In addition, we also observed 4 weeks after MI an increase of the mature and thick collagen (Figure 6b), suggesting a less IG3 uptake at that time because of the larger presence of thick collagen fibers that might be preventing the binding of IG3 to collagen molecules and/or any other ECM component. Although no significant correlation has been observed between IG3 uptake and collagen content, we do observe a tendency of a negative correlation between IG3 uptake and thick collagen content and a positive correlation between IG3 uptake and thin collagen content as much as in the infarct and in the whole heart of MI animals (data not shown).

### Galectin-3 and myocardial remodeling

In the last few years, several studies have focused their attention on the role of galectin-3 in cardiovascular disease, especially as a reliable biomarker of ECM turnover in HF patients [12,18,30] that correlated with the adverse outcome in HF [7,12]. Although never employed in humans, galectin-3 inhibition in animal studies has resulted in attenuation of cardiac inflammation, fibrosis, and LV dysfunction [5,31]. In these studies, the galectin-3 binding sites localized predominantly to the myocardial ECM and fibroblasts but they are absent from cardiomyocytes [15]. Inhibition of galectin-3 has also been reported to result in reduced plaque volume in apolipoprotein-E-deficient mice [32].

In the present study, 2 days before *ex vivo* SPECT imaging, animals underwent cardiac magnetic resonance for the assessment of LV function and infarct size by LGE; all MI animals presented similar ejection fraction and infarct size. We observed a positive correlation between IG3 uptake in the apical region and the infarct size using LGE by CMR.

### Molecular imaging of galectin-3

To our knowledge, this is the first attempt to use galectin-3 for *in vivo* molecular imaging of the heart. In another targeting attempt, the role of cardiac galectin-3 in TGRmRen2-27 (Ren-2) rats was studied wherein recombinant or biotinylated galectin-3 was pumped into the rat pericardium over a period of 4 weeks [15] and galectin-3 was localized to the areas of fibrosis in colocalization with cardiac fibroblast proliferation, collagen production, and macrophage activation.

Such data and the one here reported support the credibility of galectin-3 as an imaging tool in myocardial remodeling and HF. However, in our case, improvement of labeling strategies must be done in order to enhance IG3 heart uptake and therefore to get better signal for *in vivo* imaging.

## Conclusions

The present study reveals the potential of galectin-3 labeling to assess the myocardial remodeling process after MI. However, since IG3 heart uptake was not substantial enough for *in vivo* imaging at this stage and although IG3 appears to be an attractive targeting agent, signal enhancing strategies need to be developed if this agent wants to be translated into a clinically useful imaging agent.
